# Sexual dimorphic impact of adult‐onset somatopause on life span and age‐induced osteoarthritis

**DOI:** 10.1111/acel.13427

**Published:** 2021-07-09

**Authors:** Sher Bahadur Poudel, Manisha Dixit, Gozde Yildirim, Jose Cordoba‐Chacon, Manuel D. Gahete, Ikeno Yuji, Thorsten Kirsch, Rhonda D. Kineman, Shoshana Yakar

**Affiliations:** ^1^ Department of Molecular Pathobiology David B. Kriser Dental Center New York University College of Dentistry New York NY USA; ^2^ Section of Endocrinology, Diabetes, and Metabolism Department of Medicine University of Illinois at Chicago Chicago IL USA; ^3^ Research and Development Division Jesse Brown VA Medical Center Chicago IL USA; ^4^ Barshop Institute for Longevity and Aging Studies UTHSCSA San Antonio TX USA; ^5^ Department of Orthopaedic Surgery NYU Grossman School of Medicine New York NY USA; ^6^ Department of Biomedical Engineering NYU Tandon School of Engineering New York NY USA

**Keywords:** articular cartilage, growth hormone, health span, life span, osteoarthritis, osteophyte, subchondral bone

## Abstract

Osteoarthritis (OA), the most prevalent joint disease, is a major cause of disability worldwide. Growth hormone (GH) has been suggested to play significant roles in maintaining articular chondrocyte function and ultimately articular cartilage (AC) homeostasis. In humans, the age‐associated decline in GH levels was hypothesized to play a role in the etiology of OA. We studied the impact of adult‐onset isolated GH deficiency (AOiGHD) on the life span and skeletal integrity including the AC, in 23‐ to 30‐month‐old male and female mice on C57/BL6 genetic background. Reductions in GH during adulthood were associated with extended life span and reductions in body temperature in female mice only. However, end‐of‐life pathology revealed high levels of lymphomas in both sexes, independent of GH status. Skeletal characterization revealed increases in OA severity in AOiGHD mice, evidenced by AC degradation in both femur and tibia, and significantly increased osteophyte formation in AOiGHD females. AOiGHD males showed significant increases in the thickness of the synovial lining cell layer that was associated with increased markers of inflammation (IL‐6, iNOS). Furthermore, male AOiGHD showed significant increases in matrix metalloproteinase‐13 (MMP‐13), p16, and β‐galactosidase immunoreactivity in the AC as compared to controls, indicating increased cell senescence. In conclusion, while the life span of AOiGHD females increased, their health span was compromised by high‐grade lymphomas and the development of severe OA. In contrast, AOiGHD males, which did not show extended life span, showed an overall low grade of lymphomas but exhibited significantly decreased health span, evidenced by increased OA severity.

## Graphical abstract

In humans, the natural decline in growth hormone (GH) secretion during aging, termed somatopause, associates with reduced health span. We found that age‐induced somatopause in mice increased primary osteoarthritis (OA) of the knee joint. This was evidenced by the loss of articular cartilage, increased osteophyte formation, and thickening of the synovial membrane. At the cellular level, somatopause increased chondrocyte expression of matrix‐degrading enzymes, senescence, and inflammatory markers.

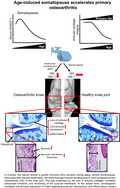

## 1 INTRODUCTION

The hypothalamic/pituitary somatotropic axis plays major roles in determining body size, body composition, and metabolic homeostasis (reviewed in [Le Roith et al., 2001]). The activity of the two systemic somatotropic factors, growth hormone (GH) and its downstream effector, insulin‐like growth factor (IGF‐1), peak during pubertal growth and decline thereafter. Pituitary GH is the main regulator of serum IGF‐1, which is produced and secreted by the liver. Both hormones have receptors in virtually all tissues and act in an endocrine and autocrine/paracrine manner. Aging associates with a spontaneous decline in GH secretion by approximately 15% for every decade of adult life (Iranmanesh et al., 1991). During middle age and after, about 35% of men are found to be GH deficient (GHD) (Rudman et al., 1990). Reductions in GH lead to a progressive drop in serum IGF‐1 levels, a state termed somatopause (Hersch & Merriam, 2008). In humans, congenital inactivation of the GH receptor associates with major reductions in pro‐aging signaling, cancer, and diabetes (Guevara‐Aguirre et al., 2011). In animal models, in addition to decreased tumor incidence and metabolic abnormalities, germline (congenital) deficiency in GH/IGF‐1 exhibits extended life span (Ikeno et al., 2009). However, other studies have shown that age‐induced somatopause, specifically in humans, associates with cognitive dysfunction, sarcopenia, osteopenia, and immune dysfunction (Doessing et al., 2010; Sathiavageeswaran et al., 2007; Sonntag et al., 1999). The discrepancy between these two observations, which on one hand shows that somatopause increases life span, but on the other hand decreases health span, has been a matter of debate for many years (Colon et al., 2019).

The GH/IGF‐1 axis plays a major role in skeletal acquisition during growth, including stimulation of osteoprogenitor differentiation and skeletal cell (osteoblasts, osteoclasts, and chondrocyte) function. The effects of GH and IGF‐1 on chondrocyte proliferation and function in the growth plate and their association with linear bone growth during development are well established. In vivo studies showed that both hormones have distinct and overlapping effects on growth plate chondrocytes (Wang et al., 2004). While GH affects chondrocytes at the germinal, proliferating, and hypertrophic zones, IGF‐1 affects mostly the proliferating chondrocytes and plays essential roles in calcification of the cartilage in chondro‐osseous junction in the growth plate (Wang et al., 2014). Very little, however, is known about the role of GH/IGF‐1 axis in joint homeostasis.

During aging, in addition to drops in GH secretion and systemic IGF‐1 levels, the pool of IGF‐1 in the bone matrix reduces and associates with bone loss and compromised bone integrity. In the articular cartilage (AC), the GH/IGF‐1 axis stimulates chondrocyte proliferation, synthesis of proteoglycans, and type II collagen, (which are the main components of the cartilage matrix), and exerts inhibitory effects on matrix‐degrading enzymes. Aging associates with significant loss in chondrocyte anabolic activity, and degradation of the extracellular matrix (ECM) (Lotz & Loeser, 2012). These changes promote excessive fibrillation, cross‐linking of collagen molecules, stiffness, reduced tensile strength, and susceptibility to fatigue failure, all contributing to increased incidence of osteoarthritis (OA), a disease of the synovial joints (Verzijl et al., 2002). OA affects the entire joint and is characterized by cartilage loss, formation of osteophytes, subchondral bone changes, and synovitis, leading to chronic pain and disability (Hunter & Bierma‐Zeinstra, 2019). Existing therapies mostly alleviate OA pain, but do not slow down the progression of OA. As detailed below, age‐induced somatopause in our mouse model resulted in macroscopic alterations in the knee joints of both male and female mice. The current study is focused on the development of primary OA in the knee joint in response to somatopause.

**FIGURE 1:**
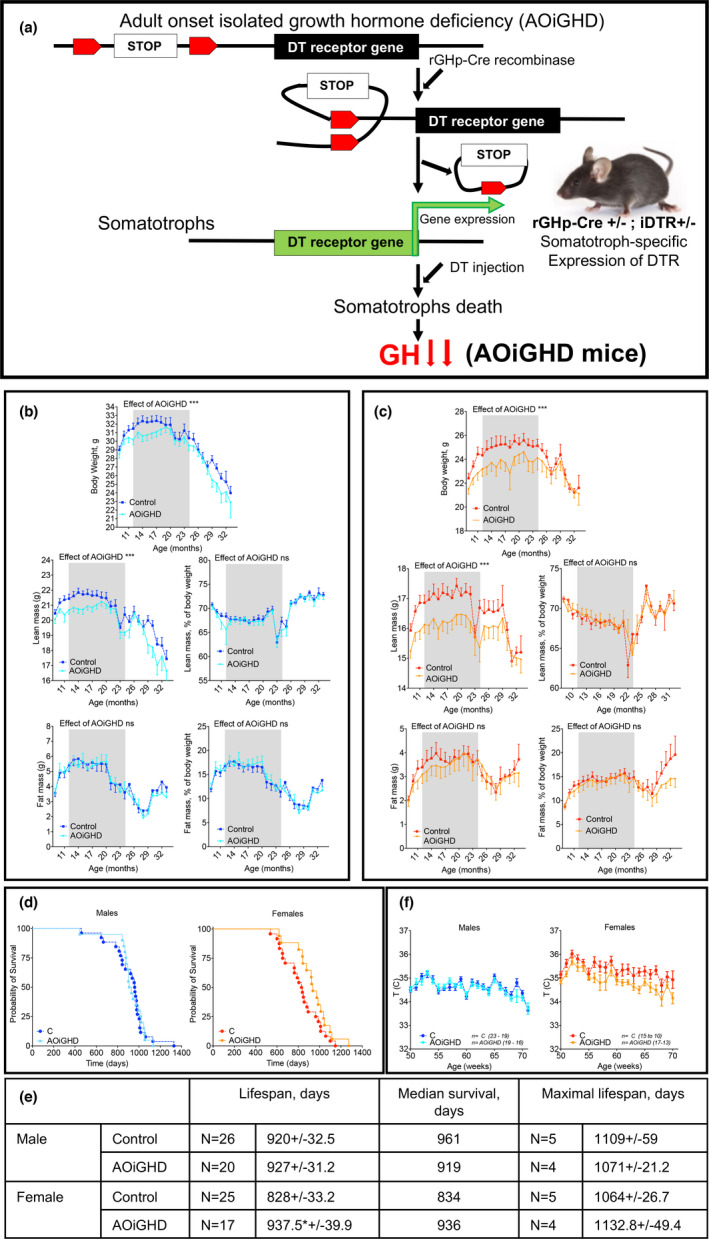
Effects of adult‐onset isolated growth hormone deficiency (AOiGHD) on NMR‐based body composition and life span. (a) AOiGHD model is based on the expression of a Cre‐recombinase under the rat GH promoter (rGHp), which releases a stop‐cassette upstream of a diphtheria toxin (DT) receptor (DTR) transgene in GH expressing somatotrophs in the pituitary. Upon DT injection, somatotrophs expressing DTR (rGHp‐Cre^+/−^; DTR^+/−^) are programmed for death, resulting in dramatic reductions in GH secretion and subsequently AOiGHD. (b) Male and (c) female mice were followed for body weight and body composition longitudinally. Body composition was examined monthly from 8 to 33 months by NMR. Presented are body weights, absolute and relative lean and fat mass, in male and female mice. (d) Life span was determined in male and female mice using Kaplan–Meier test. Sample size; male control *n* = 26, male AOiGHD *n* = 20, female control *n* = 25, female AOiGHD *n* = 17. Number of mice in each group dropped with age. (e) Kaplan–Meier data table showing life span, median survival and maximal life span of mice. (f) Rectal temperatures were monitored between 50 and 70 weeks of age in control male (*n* = 19–23), AOiGDH male (*n* = 16–19), control female (*n* = 10–15), and AOiGDH female mice (*n* = 13–17). Values are given as mean ± SEM; and *p < 0.05, **p < 0.01, and ***p < 0.001

Mice, similar to humans, develop primary OA with age. C57BL/6 mice show increased chondrocyte death and decreased AC thickness at 23 months compared to 4.5 months of age (McNulty et al., 2012). Findings in the long‐lived male Ames dwarf (Ewart et al., 2020) and Snell dwarf (Silberberg, 1972) mice (which both lack GH) have shown reductions in AC necrosis and reduced age‐associated OA, respectively. However, these congenital dwarf models show significant differences in body composition, energy metabolism, and cage activity when compared to controls, and they do not mimic the somatopause seen in humans. We hypothesized that age‐induced somatopause (reductions in GH and IGF‐1 levels) will further impair articular chondrocyte function and consequent loss of AC. To test our hypothesis, we used a mouse model of adult‐onset, isolated GH deficiency (AOiGHD), as opposed to congenital models of GH deficiency (Figure 1a). The AOiGHD mouse model is based on Cre‐dependent expression of the diphtheria toxin (DT) receptor (DTR) in somatotrophs (GH‐promoter driven Cre recombinase, rGHp‐Cre) (Luque et al., 2011). We have previously shown that administration of DT to adult rGHp‐Cre, iDTR mice resulted in the selective destruction of DTR‐expressing somatotrophs leading to a sustained reduction in GH to 20%–30% of age‐matched controls, and IGF1 levels to 75% of age‐matched controls, in both male and female mice, associated with improved insulin sensitivity without detectable impact on lactotrope, corticotrope, thyrotrope, or gonadotrope function (Cordoba‐Chacon et al., 2014; Gahete et al., 2013). We report here data on body weight, body composition, gross pathology, and life span of AOiGHD male and female mice with detailed analysis of the AC and subchondral bone in the knee joints.

## 2 RESULTS

### 2.1 AOiGHD did not have significant effect in NMR‐based body composition

AOiGHD was induced by administration of DT at 3 months of age and body composition (lean, fat, and fluid mass) assessed longitudinally from 8 to 33 months of age. In both male and female control and AOiGHD mice, body weights plateaued at ~12 months of age, remained relatively constant up to 20 months of age, and thereafter declined (Figure 1b,c). Overall, reductions in body weight (*p* < 0.0001) were more prominent in AOiGHD females as compared to AOiGHD males. Reduced body weight in AOiGHD females was associated with a greater reduction in lean mass (Figure 1b,c) as was previously shown (Cordoba‐Chacon et al., 2014). However, no differences were detected by NMR‐based fat mass (Figure 1b,c) between controls and the AOiGHD mice within sex.

### 2.2 AOiGHD increased life span in female mice

Life span was analyzed using the Kaplan–Meier method (Figure 1d). Using a sample size of 88 mice, we found that the mean life span of AOiGHD male did not differ from control mice (920 ± 32 days in controls vs 927 ± 31 days in AOiGHD). Similarly, there were no differences in median survival (961 days in controls vs 919 days in AOiGHD) or maximal life span (1109 ± 59 days in controls vs 1071 ± 21 days in AOiGHD) (Figure 1e). In contrast, AOiGHD female mice showed significantly increased mean life span (828 ± 33 days in controls vs 937 ± 40 days in AOiGHD). However, median survival (834 days in controls, vs 936 days in AOiGHD) or maximal life span (1064 ± 27 days in controls vs 1133 ± 49 days in AOiGHD) did not reach significance (Figure 1e). In our cohort, the mean life span of control males was increased as compared to control females. The difference in life span between male and female AOiGHD mice, however, did not exist. Female, but not male, AOiGHD mice showed significant (*p* < 0.0001) decrease in rectal temperature, as compared to controls (Figure 1f).

**FIGURE 2:**
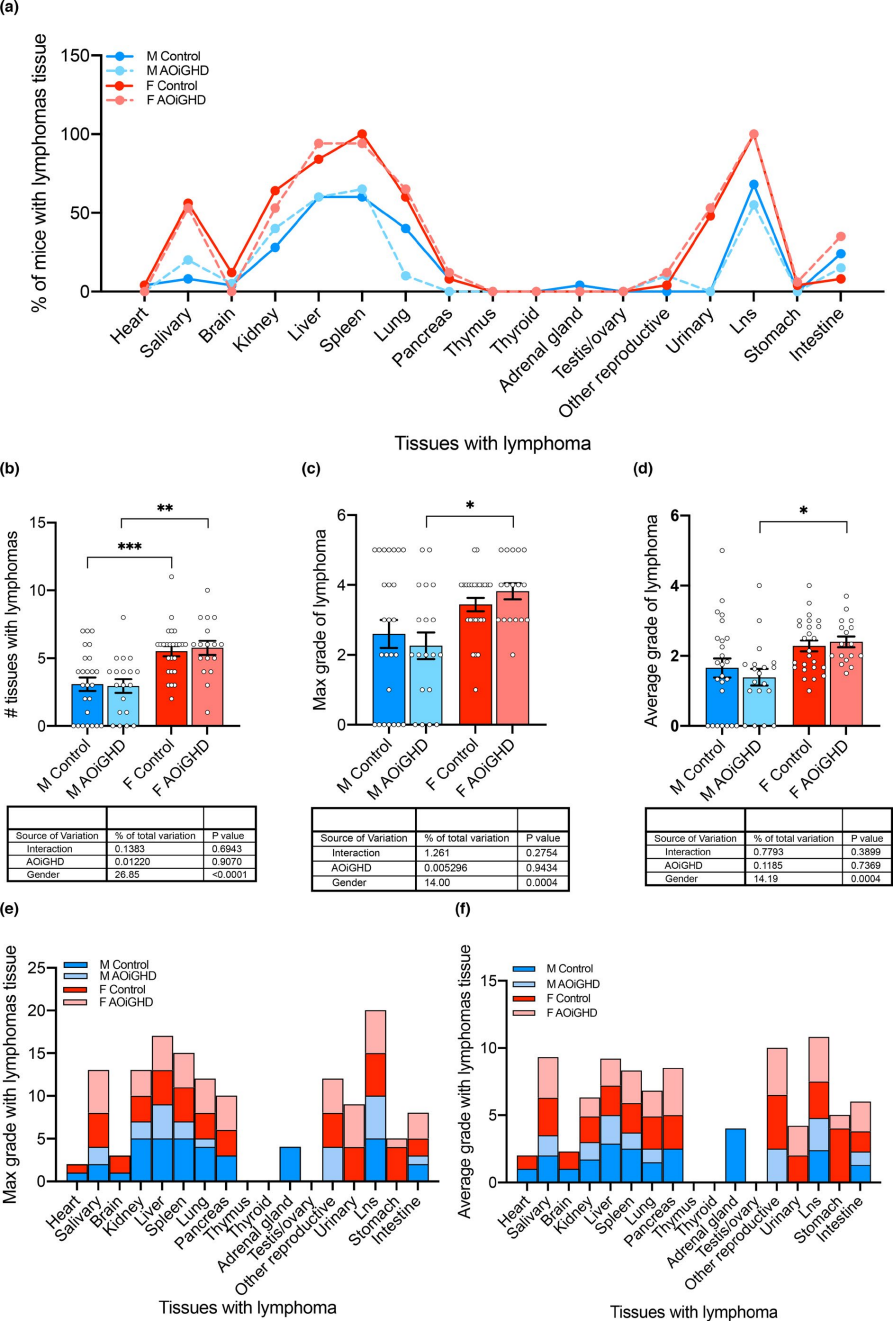
End‐of‐life pathology. Pathology examination was done for 86 mice (control male (*n* = 25), AOiGDH male (*n* = 19), control female (*n* = 25), and AOiGDH female mice (*n* = 17)). (a) Percent of mice with lymphomas in the various tissues did not differ significantly between controls and AOiGHD mice. (b) Total number of tissues with lymphomas, (c) number of tissues with max grade lymphomas, or (d) number of tissues with average grade lymphomas were significantly higher in female than male mice but did not vary with genotype. When analyzed by tissue, control males (dark blue) showed increased (e) max grade and (f) average grade lymphomas (specifically in kidney, liver, spleen, pancreas, lung, adrenal glands and lymph nodes; Lns). Data are presented as mean ± SEM, *p < 0.05, **p < 0.01, and ***p < 0.001. M and F indicate male and female, respectively

Lymphomas are very common in aged C57BL/6 mice. Approximately 70%–75% of ad libitum fed mice die of cancer and 85%–90% of the cancer is lymphoma (Ikeno et al., 2005). Pathological examinations revealed no significant differences between controls and AOiGHD mice in the percent of overall lymphomas in all tissues (Figure 2a). Overall, female mice had a higher number of tissues with lymphomas (Figure 2b) and a higher maximal grade of lymphomas than male mice (Figure 2c,d). These endpoints were not impacted by AOiGHD in female mice. However, fewer AOiGHD males developed high‐grade lymphomas (>3), with most notable reductions in the kidney, spleen, and lung than control male mice (Figure 2e,f).

### 2.3 AOiGHD associated with increased AC degradation and osteophyte formation in male mice

**FIGURE 3:**
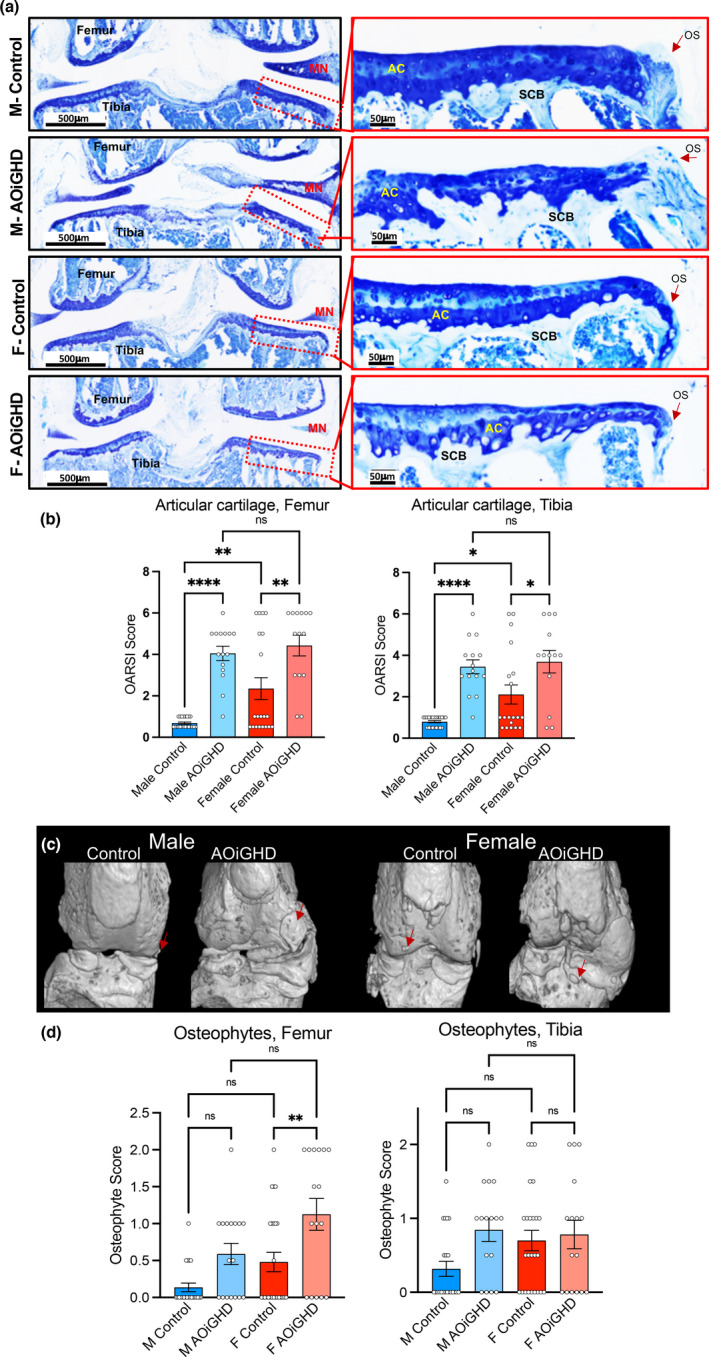
AOiGHD males show significant degradation of the AC and increases in osteophyte formation. (a) Representative toluidine blue stained sections of the knee joint. AC: articular cartilage, L: ligament, MN: meniscus, and OS: osteophyte. (b) Cartilage loss was quantified by OARSI scoring system in femur and tibia (control male (*n* = 22), AOiGDH male (*n* = 15), control female (*n* = 19), and AOiGDH female mice (*n* = 13)). (c) micro‐CT 3D images of the knee joint, osteophytes are indicated by red arrows. (d) Osteophytes were quantified in femur and tibia from toluidine blue stained sections according to the OARSI scoring system (control male (*n* = 22), AOiGDH male (*n* = 15), control female (*n* = 20), and AOiGDH female mice (*n* = 14)). Data are presented as mean ± SEM; ^ns^ non‐significant, *p < 0.05, **p < 0.01, and ***p < 0.001. M and F indicate male and female, respectively

Histological analyses were done on one randomly selected intact hind limb per mouse (a total of 87 knee joints were processed). Sections were selected based on similar anatomic depth. Our histological analysis of the knee joints revealed increased OA severity in female control compared to male control mice. Both male and female AOiGHD mice showed significantly increased OA severity compared to their respective controls, which was evidenced in both femur and tibia by decreased toluidine blue staining and markedly increased cartilage loss (Figure 3a,b).

Prior to histological analysis of the knee joints, dissected hind limbs were scanned by mCT. Out of 87 mice, we harvested two intact knee joints from 45 mice and one knee joint per mouse from the remaining 42 mice (a total of 132 knee joints were scanned by mCT). Based on 2D and 3D reconstruction of mCT scan images of the 45 mice scanned for both legs, 50% of control males, 87.5% of AOiGHD males, 87.5% of control females, and 100% of AOiGHD females developed macroscopic, radiographically visible, abaxial calcified osteophytes in both knee joints (Figure S1). Consequently, we scored osteophyte size and maturity using toluidine blue‐stained histological sections of knee joints according to previously published studies (Kaneko et al., 2013). We found that osteophyte formation was increased in aged AOiGHD males compared to control males in both femur and tibia; however, this did not reach significance (Figure 3c,d). AOiGHD females showed significantly more osteophyte formation in the femur than control female mice (Figure 3c,d).

**FIGURE 4:**
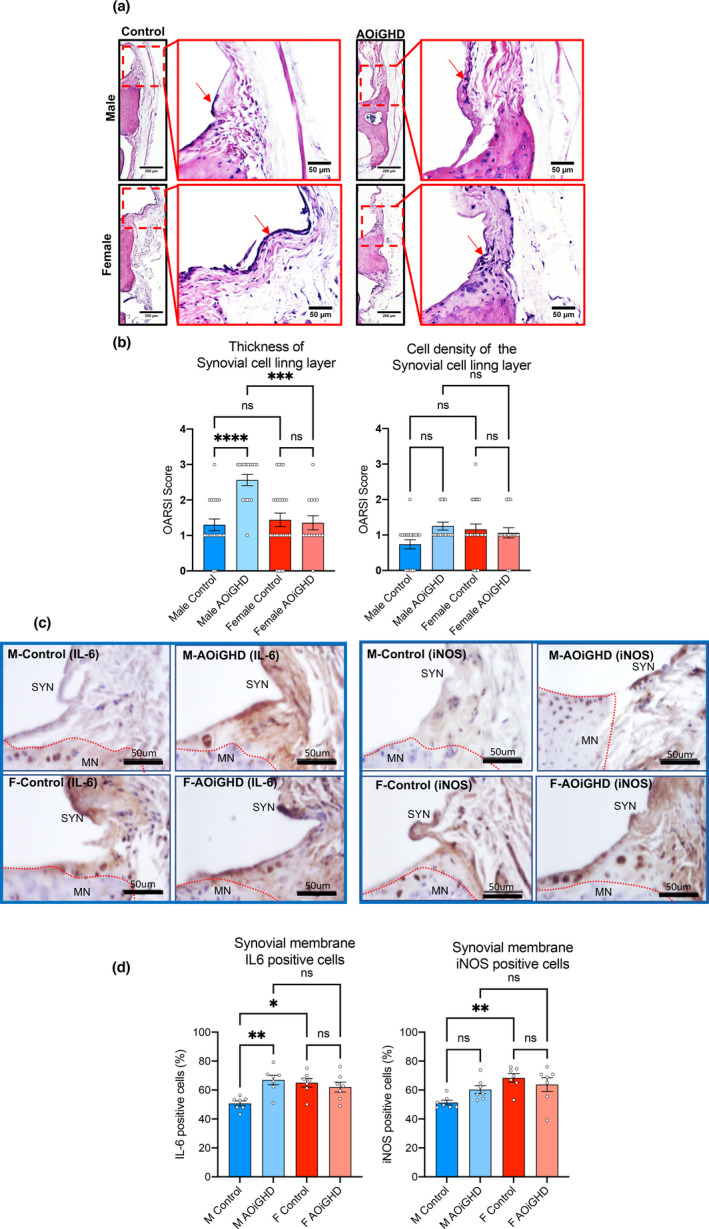
AOiGHD males show significant increases in synovitis (a) Representative H&E‐stained sections of the knee joint. Red arrows indicate the synovial membrane. (b) The thickness and cell density of the synovial cell lining layer were quantified by OARSI scoring system (control male (*n* = 21), AOiGDH male (*n* = 17), control female (*n* = 25), and AOiGDH female mice (*n* = 16)). (c) Representative images and quantification of the synovial membrane immunostained with IL6 and iNOS. MN; meniscus, SYN; synovium. (d) Synovial layer positive cells for IL6 and iNOS (control male (*n* = 7), AOiGDH male (*n* = 7), control female (*n* = 7), and AOiGDH female mice (*n* = 7)) Values given in mean ± SEM; ^ns^ non‐significant. M and F indicate male and female, respectively

Synovial inflammation or synovitis is another hallmark of OA. We analyzed synovitis in our mouse models using H&E‐stained sections (Figure 4a) according to a previously described scoring system. In this system, the thickness of the synovial cell lining layer and the cell density within this layer is scored from 0 to 3 with 0 being the thinnest synovial cell lining layer and lowest cell density and 3 being the thickest synovial cell lining layer and highest cell density (Kaneko et al., 2013). The synovial cell lining layer was significantly enlarged, and the cell density increased in AOiGHD male mice compared to control male mice (Figure 4b). No significant differences in thickness and cell density were observed between female control and AOiGHD mice. However, male AOiGHD mice showed significantly increased thickness of the synovial cell lining layer compared to female AOiGHD mice. Increased cell density in the synovial membrane in AOiGHD males and female groups as compared to control males did not reach significance. Consequently, randomly selected samples were immunostained with the inflammatory markers, IL‐6 and iNOS. IL‐6‐ and iNOS‐positive cells in the synovium significantly increased in control females as compared to control males. IL‐6‐positive cells in the synovium significantly elevated in AOiGHD male mice compared to control males. Females showed overall increases in IL‐6‐ and iNOS‐positive cells compared to male mice, but there was no effect of AOiGHD.

### 2.4 AOiGHD did not affect the height, volume, or BMD of the subchondral bone plate.

**FIGURE 5:**
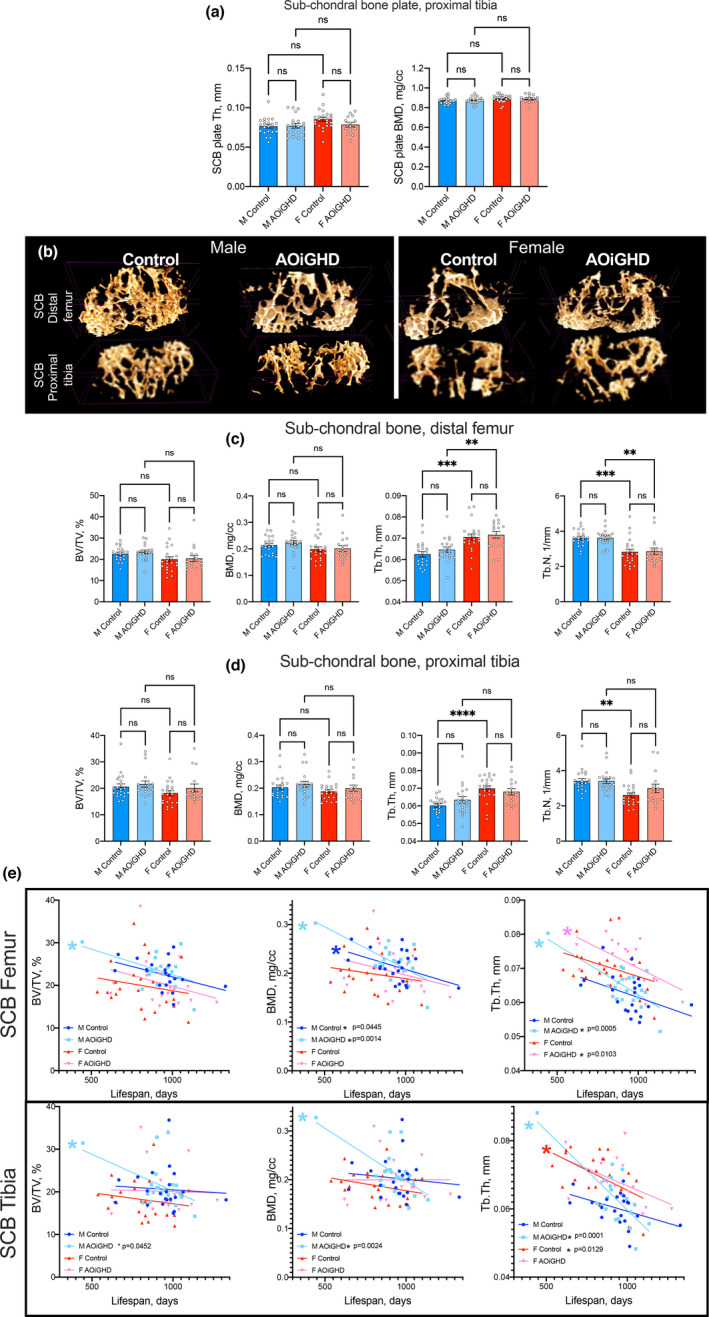
AOiGHD does not affect the thickness or mineral density of the SCB plate. (a) The thickness and mineral density of the SCB plate of the tibia (LMTP) was quantified using mCT. (b) Representative 3D images of SCB at the distal femur and proximal tibia. (c) Quantification of SCB volume/total volume (BV/TV), bone mineral density (BMD), trabecular thickness (Tb. Th), and trabecular number (Tb.N) at the distal femur and (d) proximal tibia. (e) SCB parameters in the distal femur and proximal tibia as a function of age. Values given in mean ± SEM; ^ns^ non‐significant. M and F indicate male and female, respectively (control male (*n* = 21), AOiGDH male (*n* = 20), control female (*n* = 23), and AOiGDH female mice (*n* = 15)

Previous studies have reported that the severity of OA is associated with subchondral bone (SCB) changes (Burr, 1998). The thickness of the SCB‐plate was assessed in the lateral and medial tibial plateau (LMTP) using mCT (Figure 5a). This site was chosen because it was found to be most frequently affected in age‐related OA (McNulty et al., 2011) and because the anatomical morphology of this site is easier to analyze. Micro‐CT data revealed no differences in SCB plate thickness (Th), volume (data not shown), or bone mineral density (BMD) between controls and AOiGHD mice in both sexes.

SCB volume of femur and tibia was analyzed using 3D images of micro‐CT scans (Figure 5b). We found no differences in the SCB volume/total volume (BV/TV) or in SCB BMD, in femur (Figure 5c). However, the thickness of the trabeculae (Tb. Th) was increased in control and AOiGHD female as compared to their respective males. Trabecular numbers (Tb.N), on the other hand, were decreased in control and AOiGHD female mice compared to their respective males. Similarly, in tibia no differences were detected in the SCB BV/TV or in the SCB BMD (Figure 5d).

Since life span of each mouse differed, we corrected the SCB parameters to their age at death. We found that male AOiGHD mice showed significant age‐associated declines in BV/TV, BMD, and Tb. Th in the femoral SCB, and declines in BMD and Tb. Th in the SCB of the tibia (Figure 5e). In contrast, female mice did not show reductions in any of those parameters with age. However, we found that AOiGHD female mice showed declines in Tb. Th in femoral SCB with age (Figure 5e).

Analyses of cortical bone at the femur mid‐diaphysis and trabecular bone at the femur distal metaphysis did not reveal significant differences between genotypes in both sexes (Figure S2a,b).

### 2.5 AOiGHD is associated with increased chondrocyte senescence and inflammation.

**FIGURE 6:**
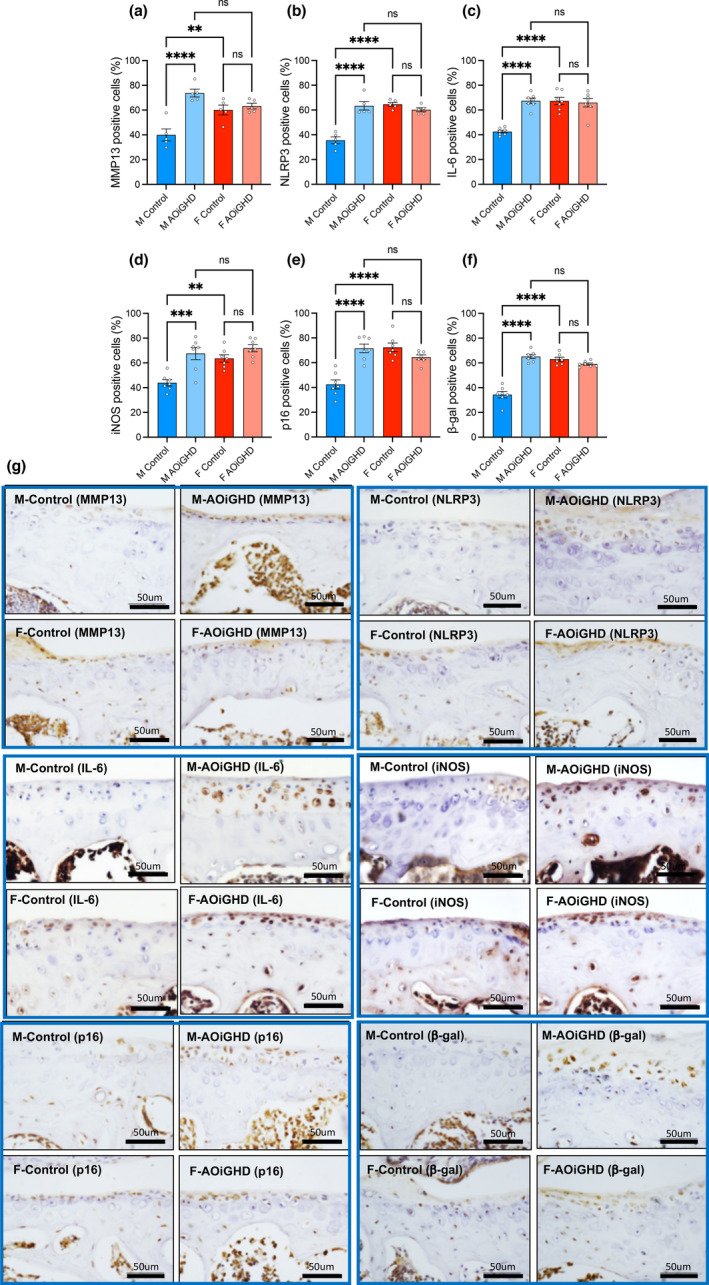
AOiGHD male mice show increased inflammation and senescence in the AC. (a) MMP‐13, (b) NLRP3, (c) IL6, (d) iNOS, (e) p‐16, and (f) β‐galactosidase. Data presented as mean ± SEM; ^ns^ non‐significant, *p < 0.05, **p < 0.01, and ***p < 0.001. M and F indicate male and female, respectively (control male (*n* = 5–7), AOiGDH male (*n* = 5–7), control female (*n* = 5–7), and AOiGDH female mice (*n* = 5–7)). (g) Representative images of the articular cartilage immunostained with the aforementioned antibodies. Scale bar, 50 µm

To gain insight into the molecular mechanism underlying the pathogenesis of OA in aged mice, we performed immunohistochemistry of several predominant markers (Loeser et al., 2016). Specifically, we found increases in the matrix‐degrading enzyme, matrix metalloproteinase‐13 (MMP13) immunopositive articular chondrocytes in AOiGHD male knee joint as compared to control males, indicating increased cartilage degradation (Figure 6a, Figure S3). The inflammatory marker, pyrin domain containing 3 (NLRP3) inflammasome, known to significantly increase in OA (McAllister et al., 2018), indicated approximately 35% of NLRP3‐positive chondrocytes in male controls (Figure 6b), while the rest of the groups showed significantly higher (60%) number of NLRP3‐positive chondrocytes. Immunopositive articular chondrocytes for IL6 (Figure 6c) and iNOS (Figure 6d) increased in AOiGHD male as compared to control male mice. Finally, we found significant increases in the number of p16 (Figure 6e) and β‐galactosidase (Figure 6f) positive cells, in AOiGHD male in both femur and tibia as compared to male controls, indicating increased senescence. Female mice showed overall increases in MMP‐13, NLRP3, IL‐6, iNOS, p16, and β‐galactosidase‐positive cells compared to male mice, but there was no effect of AOiGHD.

## 3 DISCUSSION

Our study demonstrates that induction of GHD in adult mice leads to various sex‐dependent phenotypes. In females, adult‐onset GHD (AOiGHD) increased mean life span, but was associated with reduced health span evidenced by loss of lean body mass in middle age mice, increased neoplastic lesions, and development of severe OA. In males, induction of GHD did not impact life span, it reduced lean body mass in middle age mice, was associated with development of severe OA, but tended to reduce the number and grade of lymphomas within tissue type. Additionally, while control males showed increased mean life span as compared to control females, this difference was not found in the AOiGHD mice. Extension of maximal life span in females, but not males, was observed in a mouse model of adult‐onset disruption of the GHR (Junnila et al., 2016). These sex differences may be related to the impact of GHD or GH insensitivity on IGF1, since various mouse models with a reduction in IGF1 or IGF1 sensitivity show female‐specific life span extension (Yuan et al., 2020). Nevertheless, as previously reported (Austad & Fischer, 2016), there is no consistent sex difference in mouse longevity. The conditions favoring survival of one sex over the other are site‐dependent and currently unknown in mice. Further, we cannot exclude the possibility that the lack of significant differences in life span in AOiGHD male mice as compared to controls may result from a limited sample size. Importantly, emphasizing longevity metrics alone as an endpoint, assuming that health span correlates directly with life span, is not always true. In humans, females live longer than males, but have a greater age‐related morbidity by a number of measures (Austad & Fischer, 2016). In the current study, we also found dichotomy between life span and health span in AOiGHD female mice. While AOiGHD female lived longer, they had comparable burden of lymphomas and more severe OA as compared to control females. Clearly, factors such as GH‐dependent stress resistance, programmed during puberty, may uncouple life span and health span; however, these were not addressed in the current study. Finally, similar to females with hemizygous loss of the IGF1R (*Igf1r* ^−/+^ mice) (Xu et al., 2014), the increase in life span of AOiGHD female mice was associated with a reduction in resting (daytime) body temperature (Cintron‐Colon et al., 2017). As previously reviewed (Conti, 2008), small reductions in body temperature are associated with increased longevity in a variety of animal species and in models with defects in GH production and signaling. However, the mechanisms linking body temperature to longevity in our model remain to be determined.

Our study shows that control female mice developed more severe primary OA than control male mice. Similar findings in humans have shown that females are more prone to OA and their disease progression is more severe, especially after menopause (Srikanth et al., 2005). It is still, however, unclear why women after menopause are more susceptible to OA development with a faster progression. Several studies have tested the hypothesis that sex steroid hormones may play a role in OA progression. However, data from several clinical trials are conflicting, showing either presence or absence of correlation between OA and sex hormone status (Cirillo et al., 2006; Nevitt et al., 2001). In the current study, we also found that female mice (toluidine blue staining) have more severe OA, as compared to males. However, somatopause (AOiGHD) increased the severity of OA in both sexes, such that the sexual dimorphism seen between control male and female mice did not exist. A previous study has shown that intra‐articular injection of GH into knee joints of New Zealand White rabbits, in which OA was induced by collagenase injection, slowed down the progression of OA (Kim et al., 2010). Our study demonstrating that GH deficiency in adulthood accelerated OA development and progression in male and female mice confirms the potential benefit of GH in slowing down the progression of OA. However, our study indicates that potential novel therapeutic strategies for the treatment of OA have to be critically analyzed for their effects in both sexes. Notably, however, the aforementioned study used only male rabbits for the intra‐articular injection of GH (Kim et al., 2010).

Little is known about the direct effects of GH on AC homeostasis and maintenance. More, however, is known about the effects of IGF1 on articular chondrocytes. IGF1 has been shown to play an important anabolic role in AC and to have anticatabolic activities. In addition, IGF1 has been shown to stimulate matrix synthesis by chondrocytes and to inhibit some of the deleterious effects of interleukin‐1 (IL‐1) on chondrocytes (Im et al., 2003).

The progression of OA associates with increases in inflammatory signals in the synovial membrane and chondrocytes in the AC, even in the early stages of OA (reviewed in Kapoor et al., 2011 and Felson, 2006). We found that AOiGHD in males associated with thickening of the synovial cell layer and synovitis. Accordingly, NLRP3 inflammasome, iNOS, and IL‐6 were elevated in AOiGHD males in both cells of the synovial membrane and the AC. It was recently reported that aged GH receptor null mice (GHRKO) exhibit downregulation of the NLRP3 inflammasome, linking NLRP3‐mediated innate immune signaling to the increased longevity of these mice (Spadaro et al., 2016). Increases in inflammatory markers were associated with increased levels of the matrix‐degrading enzyme, MMP13, in articular chondrocytes in male AOiGHD mice as compared to control mice. Finally, chondrocyte senescence has been shown to play a major role in OA development and progression (Loeser et al., 2016). We found increased number of chondrocytes positive for the senescence markers p16 and β‐galactosidase in male AOiGHD mice as compared to control mice. AOiGHD females exhibited increased AC loss as compared to control females. However, showed comparable synovitis and similar levels of inflammation and senescence in the AC as control females, possibly suggesting that AOiGHD increases susceptibility to inflammation.

Primary OA in mice and humans show many similarities including cartilage degradation, osteophyte formation, thickening of the synovial membrane, and increased SCB volume (Vincent, 2020). The sequence of the aforementioned pathological events and the relationship between damage to the SCB, AC degradation, and inflammation remain unknown. Furthermore, primary OA does not always involve all the aforementioned characteristics. Interestingly, cartilage degradation in control and AOiGHD mice were not accompanied by SCB thickening as seen in other OA animal models, and as has been proposed to characterize human OA (Burr, 1998). Our findings are in accordance with a previous study showing that chronic GH/IGF‐1 deficiency in male rats resulted in increased severity of AC lesions without bony changes, at times seen in OA (Ekenstedt et al., 2006). These findings, together with our data, suggest that cartilage lesions, osteophytes, and synovitis can occur without the bony changes. It is possible that no SCB thickening is being observed in AOiGHD mice because of the possible requirement of GH/IGF‐1 as a mediator of increased bone formation.

Bones were analyzed from control and AOiGHD mice at time of death, which ranged from 23 to 30 months of age. At such advanced ages, mCT analyses of several skeletal sites (femur mid‐diaphysis, femur distal metaphysis, L5 vertebra (data not shown), and SCB of the tibia and femur) showed only minor differences in bone morphology that were sex dependent, and more profound in female mice, regardless of genotype. Our data are in line with studies showing that reduction in bone mineral content (BMC) and BMD is more marked in patients with childhood‐onset (CO) GHD than in patients with AO‐GHD (Tritos et al., 2012). Indeed, older GHD subjects (>65 years old) without GH replacement therapy exhibit bone mass and density that are approximately similar to that in healthy age‐matched controls (Murray et al., 2006). However, they are at a higher risk of fractures than healthy controls of the same age and can benefit from GH replacement therapy depending on age, sex, and onset of the disease (childhood versus adulthood) (Elbornsson et al., 2012).

Finally, studies in humans, as well as in animals, have shown that the GH/IGF1 axis regulates body composition and bone mineral acquisition. Congenital isolated GHD, described in a large cohort of subjects in northeastern Brazil who carried homozygous mutation in the gene for GH‐releasing hormone receptor (GHRHR) (Salvatori et al., 2001), was associated with severe growth retardation, increased body adiposity, and reduced lean body mass. However, unlike congenital (childhood) GHD, adults with GHD do not show differences in body growth parameters, but exhibit abnormal body composition (Modesto Mde et al., 2014), increased central adiposity (Weaver et al., 1995), decreased muscle mass (Janssen et al., 1999) and muscle strength (Widdowson & Gibney, 2008). We used the AOiGHD mice as a model of human adult‐onset GHD (AO‐GHD). As previously reported for this model (Luque et al., 2011), induction of AOiGHD at 3 months of age resulted in significant decrease in GH secretion and concomitant reductions in serum IGF‐1 levels. We found significant reductions in absolute lean body mass in mid‐age of both male and female AOiGHD. However, relative lean mass in AOiGHD cohorts did not differ from controls in either sex.

In summary, the current study emphasizes the importance of the GH/IGF axis in the regulation of age‐related pathology. Interestingly, adult‐onset, isolated GHD (AOiGHD) was associated with increased life span in female mice, without improvement of their health span. AOiGHD females showed lymphomas in multiple tissues and developed severe OA, despite reductions in GH/IGF‐1 levels. In AOiGHD males, we found increases in age‐related primary OA evidenced by cartilage degradation, increased osteophytes formation, and synovitis as compared to controls. OA‐related pathology in control or AOiGHD mice was not accompanied by SCB changes. We cannot exclude the possibility that the sexual dimorphism reported herein may be affected by secondary confounders that may or may not depend on the GH/IGF‐1 axis, such as muscle mass (that significantly reduced in AOiGHD mice), cage activity, and pain among others. While our studies revealed an important role of GH/IGF1 in joint homeostasis and maintenance, future studies are needed to identify GH/IGF1‐dependent molecular mediators that may be used as targets for therapeutic strategies to delay or prevent primary OA.

## 4 EXPERIMENTAL PROCEDURES

### 4.1 Animals

As previously reported ((Luque et al., 2011) and in Supporting Information), a mouse model of adult‐onset, isolated GH deficiency (AOiGHD) was generated by crossbreeding mice harboring the rat GH promoter (GHp)‐driven Cre‐recombinase (Cre) to mice harboring an inducible monkey diphtheria toxin receptor (iDTR), both in a C57BL/6 genetic background. Male and female mice, heterozygous for iDTR, with (Cre^+/−^, iDTR^+/−^) or without (Cre^−/−^, iDTR^+/−^) Cre‐recombinase, were treated with diphtheria toxin (DT, Sigma‐Aldrich) between 10 and 12 wks of age by continuous low dose delivery via Alzet osmotic minipumps (6 ng/h for 7 d), where pumps were surgically placed and removed under isoflurane anesthesia. Mice were maintained under conventional housing conditions and supplied standard rodent chow diet (fat, 17 kcal%; carbohydrate, 56 kcal%; and protein 27 kcal%—Formulab Diet, Purina Mills, Inc.) and tap water *ad libitum*, for the duration of the study. Mice were group‐housed, within litter and sex, starting at 3–4 mice/cage, with numbers falling with age due to attrition. Mice were originally maintained on corn‐cob bedding, standard for the vivarium, but due to the high incidence of age‐associated dermatitis observed in C57Bl/6 background, they were switched to a paper bedding (TeKFresh, Tekland) starting at 1 year of age. Overall, 88 mice were used in the study (*n* = 26 male controls, *n* = 20 male AOiGHD, *n* = 25 female controls, and *n* = 17 female AOiGHD). Mice were followed longitudinally from 8 months of age to end of life. Mice started to die at ~23 months of age and onwards. Animal protocol was reviewed and approved by the Institutional Animal Care and Use Committees of the University of Illinois at Chicago and the Jesse Brown VA Medical Center.

### 4.2 Body composition and survival study

Whole‐body composition was assessed monthly using NMR minispec (LF50 BCA Analyzer, Bruker) between 8 and 33 months of age. Rectal temperatures were taken between 11–18 months of age (Braintree Thermometer [digital] with mouse rectal probe). Mice were visually observed daily. Date of death was noted and mice found to be moribund (expected to die within the next 24–48 h) were euthanized by decapitation and trunk blood collected, with the date of euthanasia recorded as the date of death.

### 4.3 Pathological Examination

The majority of mice were euthanized when found severely moribund. Mice were only used for analysis if death occurred less than 12 h before analysis. All 88 mice included in the study went through pathological examination. Mice that were euthanized or died spontaneously were removed from the cage, immediately necropsied with gross pathological examination, and preserved in 10% buffered formalin. After the mice were examined for gross pathological lesions, the following organs and tissues were excised and fixed with Bouin's solution: brain, pituitary gland, heart, lung, trachea, thymus, aorta, esophagus, stomach, small intestine, colon, liver, pancreas, spleen, kidneys, urinary bladder, reproductive system (male: prostate, testes, epididymis, seminal vesicles; female: ovaries, and uterus), thyroid gland, adrenal glands, parathyroid glands, psoas muscle, knee joint, sternum, and vertebrae. Any other organ or tissue in which lesions were observed by gross inspection was excised. The fixed tissues were embedded in paraffin, sectioned at 5 μm, and stained with hematoxylin–eosin. Diagnosis of each histopathological change was made using histological classifications for aging mice, as previously described (Ikeno et al., 2005). Two pathologists separately examined the samples without knowledge of their genotype or age. The probable cause of death was determined independently by the two pathologists based on the severity of the pathology found at necropsy. In more than 90% of the cases, there was agreement by the two pathologists. In cases where the two pathologists did not agree or where disease did not appear severe enough, the cause of death was categorized as unknown.

### 4.4 Micro‐computed tomography

Micro‐computed tomography (Micro‐CT) was done in accordance with the American Society for Bone and Mineral Research (ASBMR) guidelines (Bouxsein et al., 2010). Out of 87 mice, we harvested 2 intact knee joints from 60 mice and one knee joint per mouse from the remaining 17 mice (a total of 137 knee joints were scanned by mCT). Hind limbs were dissected and fixed in 10% formalin, cleaned to remove skin and extra tissues, transferred to 70% ethanol, and stored at 4°C until the time of analysis. The left limb with intact femur and tibia including the knee joint was scanned using a high‐resolution SkyScan micro‐CT system (SkyScan 1172) containing 10‐M digital detector set at a 10W energy level (100 kV and 100 μA), with a 0.5 mm aluminum filter with a 9.7 μm image voxel size. Femur cortical bone was analyzed in a 2.0 mm femur mid‐diaphyseal region directly below the third trochanter. Measurements included total cross‐sectional area inside the periosteal envelope (T. Ar, mm^2^), cortical bone area (B. Ar, mm^2^), cortical cross‐sectional thickness (Cs. Th, mm), and bone mineral density (BMD, g/cc). Trabecular bone parameters were taken at the femur distal metaphysis in a 2.5 mm region below the growth plate and included bone volume fraction (bone volume/tissue volume, (BV/TV %), trabecular thickness (Tb. Th, mm), trabecular number (Tb.N, 1/mm), and bone mineral density (BMD, mg/cc). Likewise, subchondral bone parameters were taken in proximal tibia and distal femur below the AC avoiding cortical bone. Data reconstruction was done using NRecon software (version 1.7.3.0; Bruker micro‐CT), data analysis was done using CTAn software (version 1.17.7.2+; Bruker micro‐CT), and 3D images were done using CT Vox software (version 3.3.0 r1403; Bruker micro‐CT).

### 4.5 Histology

A total of 87 randomly selected hind limbs (one per mouse) were collected from 23 to 33 months old mice and fixed in 10% formalin. Following decalcification in 10% EDTA for 4 weeks, specimens were dehydrated using graded alcohol series and xylene. The knee joints were uniformly positioned, embedded in paraffin, and sectioned coronally at a thickness of 5–7 μm. Sections from all knee joints were stained with 1% toluidine blue and hematoxylin and eosin (H&E). A few sections that showed poor quality were excluded.

For immunostaining, 6 to 8 randomly selected sections/groups were deparaffinized by xylene, rehydrated through the descending alcohol grade series and distilled water, and treated with 3% hydrogen peroxide to block the endogenous peroxidase. The antigen retrieval was done by antigen unmasking solution following the instruction provided by the manufacturer (H‐3300; Vector Laboratories). The sections were incubated with 5% bovine serum albumin for 30 min at room temperature, then with primary antibodies to p16 (1:100; #PA30670, Invitrogen), β‐galactosidase (1:600; #ab196838, Abcam), NLRP3 (1:200; #ab214185, Abcam), MMP‐13 (1:200; #ab39012, Abcam), F4/80 (1:100, #ab6640, abcam), iNOS (1:200, #PA3‐030A, Invitrogen), IL‐6 (1:50, #bs‐0782R, Bioss), and IgG isotype control (1:200, #ab37415, abcam) overnight at 4°C and HRP conjugated secondary antibody (Cat. No. PK4001, Vector Laboratories) for 1 h at RT. Immunoactivity was detected using the horseradish peroxidase‐3,3′‐diaminobenzidine system (cat. no. cts005; R&D Systems) followed by counterstaining with hematoxylin (Sigma). The images were acquired by Aperio CS2 Scanner (Leica Biosystems) and analyzed by Fiji ImageJ (version 1.51r; NIH). Isotype control images are presented in Figure S4.

### 4.6 Osteoarthritis score

Cartilage damage at medial and lateral tibio‐femoral joints was evaluated by two blinded observers using the Osteoarthritis Research Society International (OARSI) scoring system as described in earlier studies (Kaneko et al., 2013). *Loss of cartilage proteoglycan* scored by toluidine blue staining; normal staining of non‐calcified cartilage, scored 0; Decreased but incomplete loss of toluidine blue staining over 1%–100% of the articular surface 1; Complete loss of toluidine blue staining in the non‐calcified cartilage extending to <25% of the articular surface scored 2; extending to 25%–50% of the articular surface scored 3; extending to 50%–75% of the articular surface scored 4; extending to >75% of the articular surface scored 5. *Osteophyte maturity* was scored 0 where no osteophytes were detected, 1 when osteophytes were composed of precartilaginous lesion, 2 when osteophytes were composed predominantly of cartilage, 3 when osteophytes were composed of mixed cartilage and bone, and 4 when osteophytes were composed predominantly of bone. Finally, *synovitis* was scored from H&E‐stained sections according to (Kaneko et al., 2013). Briefly, the thickness of the synovial cell lining layer and the cell density within this layer was scored from 0 to 3, with 0 being the thinnest synovial cell lining layer and low cell density and 3 being the thickest synovial cell lining layer and high cell density.

### 4.7 Statistical analysis

The data are presented as mean ± standard error of the mean (SEM). For the comparison between groups, data were analyzed by two‐way analysis of variance (ANOVA) followed by post hoc Tukey's test (GraphPad Prism version 9.0). The difference between the groups was considered statistically significant when there was *p* < 0.05.

## CONFLICT OF INTEREST

The authors declare no conflict of interest.

All authors have discussed the results and approved the final version of the manuscript. SY is the guarantor of this work and, as such, had full access to all the data in the study and takes responsibility for the integrity of the data and the accuracy of the data analysis.

## AUTHOR CONTRIBUTIONS

SBP characterized the AC morphology and histology. MD performed histology. GY acquired micro‐CT data and performed image analyses. JCC and MDG followed the mouse cohorts and collected data on body weight, body composition, and rectal temperatures and helped in summary of the pathology reports. IY performed end‐of‐life pathology. TK supervised morphology and histology studies of the AC, scored OA lesions in all samples, and helped in manuscript preparation. RDK designed and established the mouse cohorts, oversaw life span studies, end‐of‐life pathology and helped in manuscript preparation. SY oversaw all studies involving the AC and bone characterizations, summarized data and prepared the manuscript.

### Open Research Badges

This article has earned an Open Data, for making publicly available the digitally‐shareable data necessary to reproduce the reported results. The data is available at https://data.mendeley.com/datasets/rfn8jtkjd4/draft?a=e55ad3f8‐18a8‐4836‐85ef‐1ccdebc864bb and https://doi.org/10.17632/rfn8jtkjd4.1.

## Supporting information

Figure S1

Figure S2

Figure S3

Figure S4

## Data Availability

The datasets generated and analyzed during the current study are available from the corresponding author on reasonable request. Our studies do not include the use of custom code or mathematical algorithms. We have included citations for available data in the references section.
