# Supplementary figures and images for: Sexual dimorphic impact of adult‐onset somatopause on life span and age‐induced osteoarthritis
Source: Aging Cell. 2021 Jul 9;20(8):e13427. doi: 10.1111/acel.13427 (PMC8373322; doi:10.1111/acel.13427)

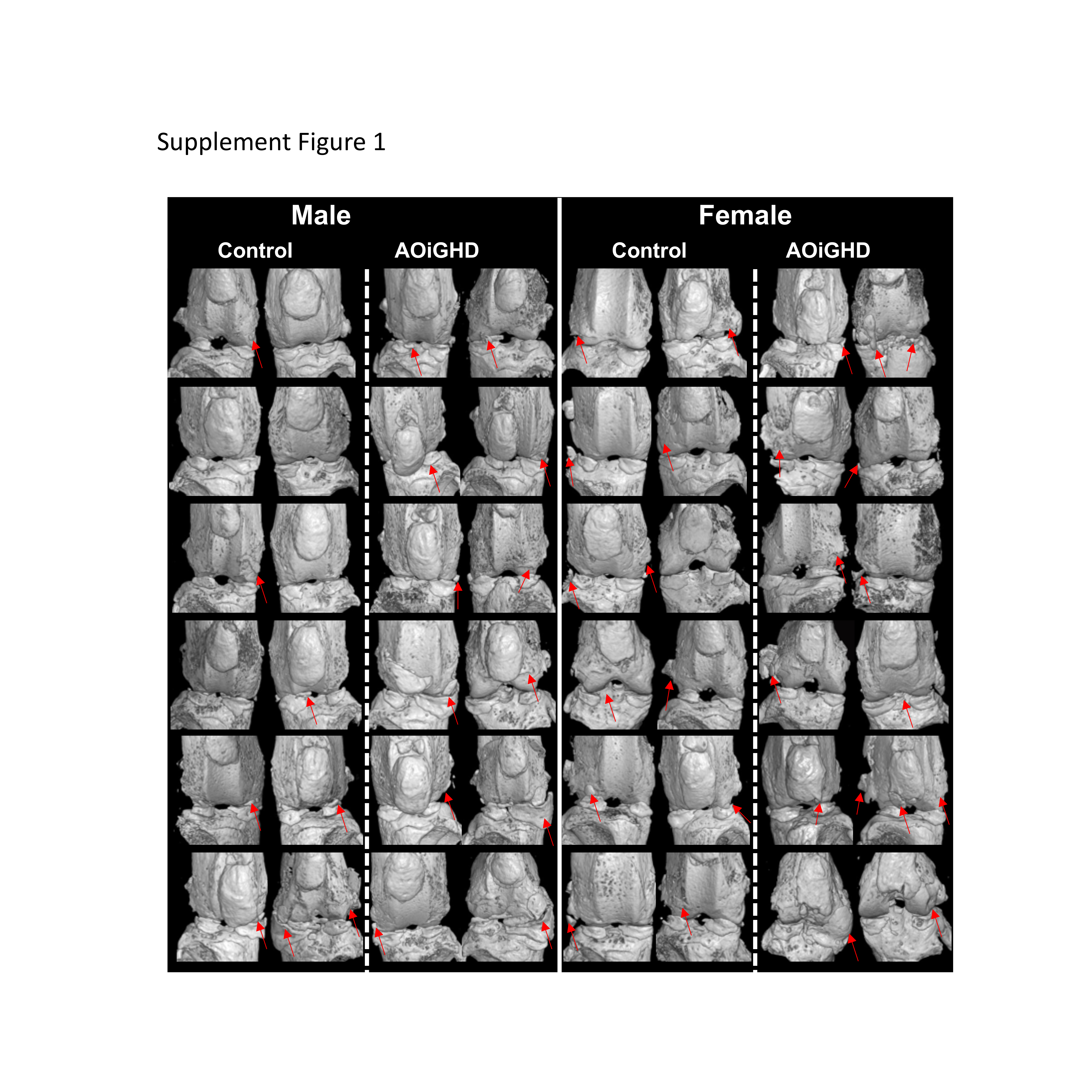

Supplement: Supplementary file 1 — Figure S1 [file ACEL-20-e13427-s003.png]

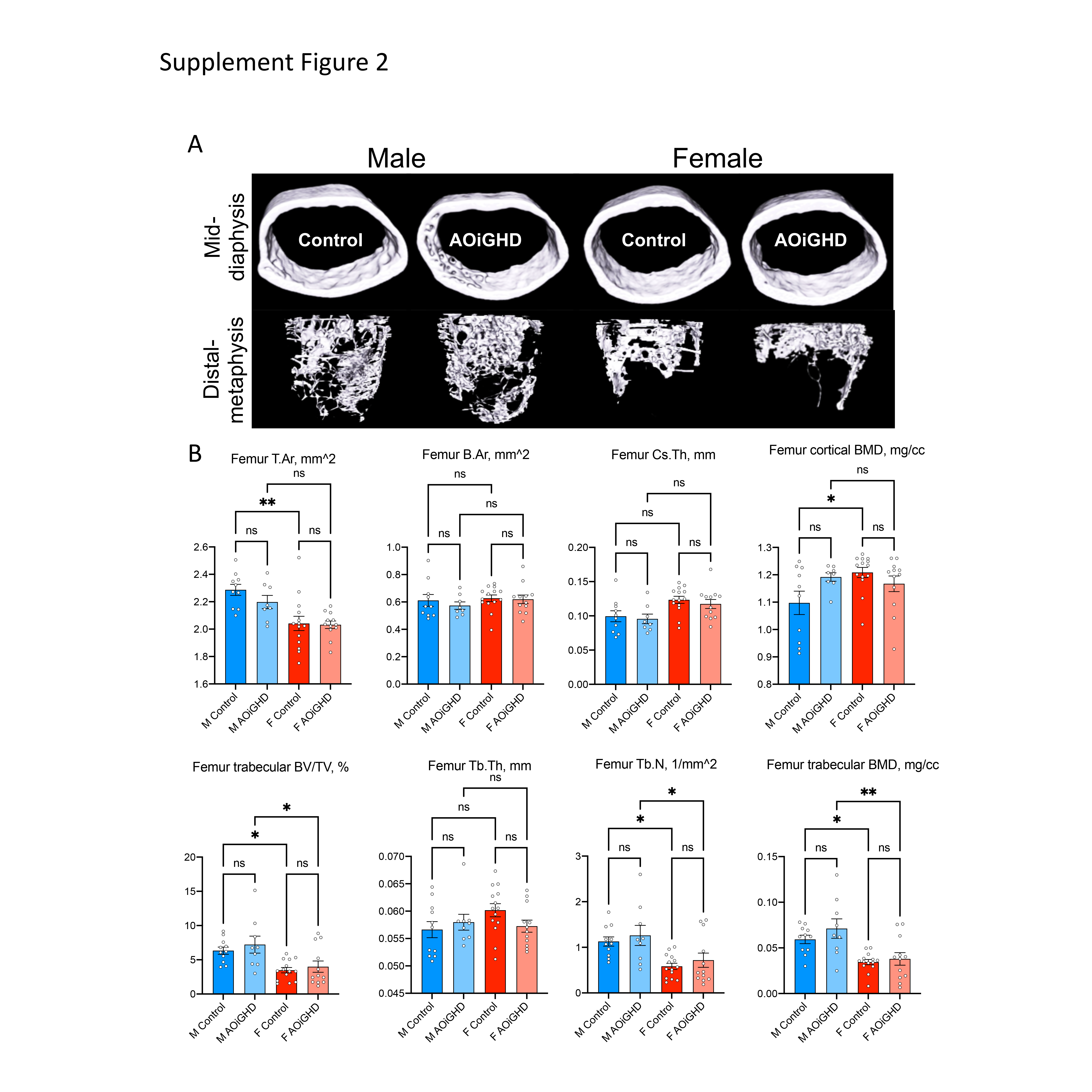

Supplement: Supplementary file 2 — Figure S2 [file ACEL-20-e13427-s002.png]

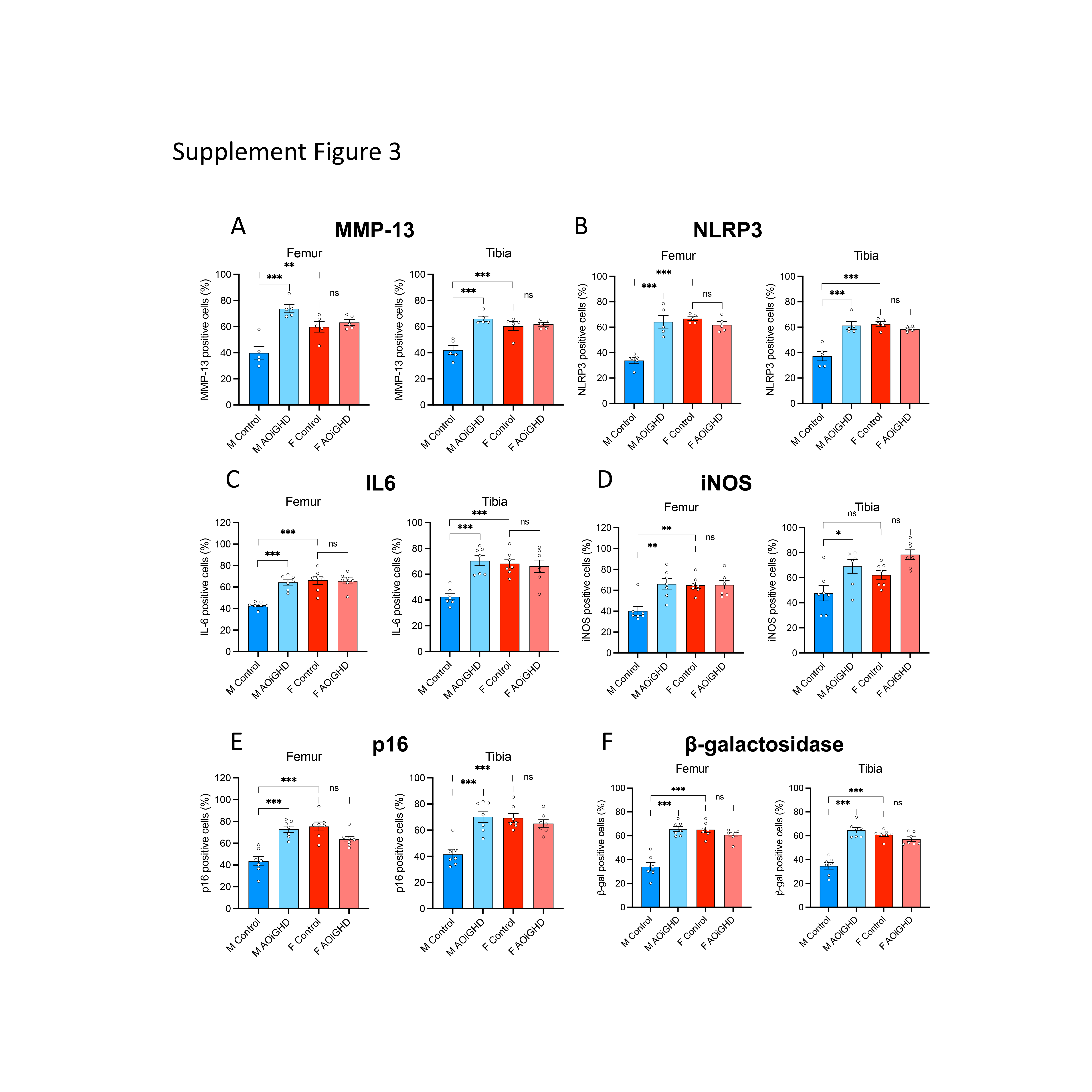

Supplement: Supplementary file 3 — Figure S3 [file ACEL-20-e13427-s004.png]

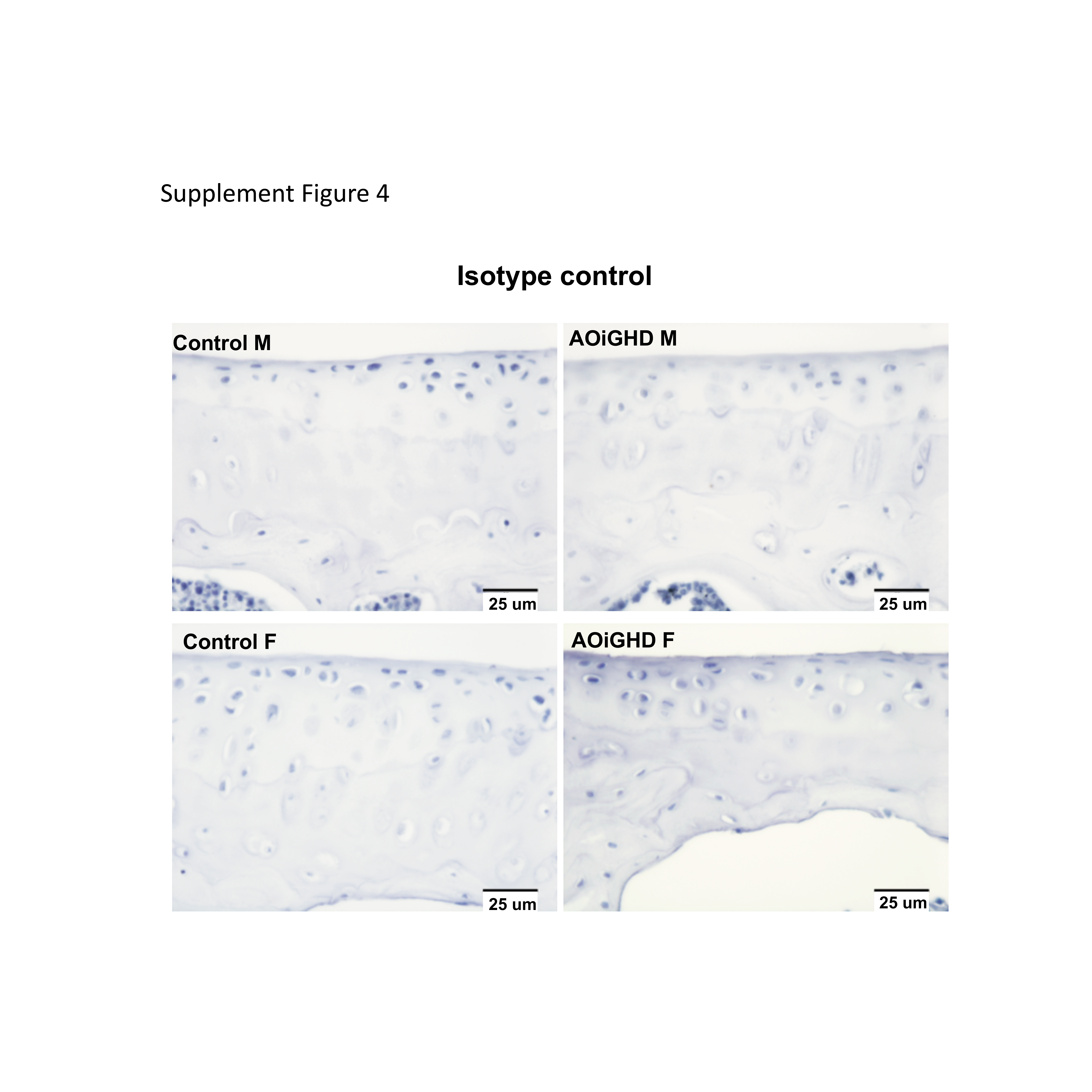

Supplement: Supplementary file 4 — Figure S4 [file ACEL-20-e13427-s001.png]
